# Hybrid Decision-Making Management for Material Selection in the Design of Wearable Pressure-Sensing Orthoses in Neurorehabilitation

**DOI:** 10.3390/biomimetics11060395

**Published:** 2026-06-04

**Authors:** Liliana-Laura Bădiță-Voicu, Roxana-Mariana Nechita, Adrian-Cătălin Voicu, Marius-Ionel Anton, Dana-Corina Deselnicu, Corina-Ionela Dumitrescu, Cristian Radu Badea

**Affiliations:** 1 Mechatronics Micro- and Nanotechnologies Department, National Institute of Research and Development in Mechatronics and Measurement Technique, 021631 Bucharest, Romania; badita_l@yahoo.com; 2 Doctoral School of Entrepreneurship, Engineering, and Business Management, National University of Science and Technology POLITEHNICA Bucharest, 060042 Bucharest, Romania; roxana.nechita2000@gmail.com (R.-M.N.); marius.anton.ionel.1985@gmail.com (M.-I.A.); 3 Department of Entrepreneurship and Management, Faculty of Entrepreneurship, Business Engineering and Management, National University of Science and Technology POLITEHNICA Bucharest, 060042 Bucharest, Romania; dana.deselnicu@upb.ro; 4 Department of Biomedical Mechatronics and Robotics, National Institute of Research and Development in Mechatronics and Measurement Technique, 021631 Bucharest, Romania; 5 Department of Mechatronics and Smart Measurement, National Institute of Research and Development in Mechatronics and Measurement Technique, 021631 Bucharest, Romania; adresacontact@gmail.com; 6 Ortotech SRL, 30573 Bucharest, Romania; 7 Department of Economics, Faculty of Entrepreneurship Business Engineering and Management, National University of Science and Technology POLITEHNICA Bucharest, 060042 Bucharest, Romania; corina.dumitrescu@upb.ro

**Keywords:** material selection, wearable orthoses, pressure-sensing, soft robotics, MCDM

## Abstract

Wearable pressure-sensing orthoses are increasingly used in neurorehabilitation to support gait recovery, monitor plantar pressure distribution, and improve patient mobility during repetitive therapy sessions. The performance of these devices depends strongly on the materials used in the skin-contact layer, since material properties influence comfort, flexibility, durability, and force transmission during daily use. This study proposes a hybrid multi-criteria decision-making framework based on the Analytic Hierarchy Process (AHP) and the VIKOR method for material selection in sensor-integrated plantar orthoses. Five candidate materials, ethylene vinyl acetate (EVA), polyethylene (PE), polyurethane (PU), cobalt–chromium–molybdenum alloy (CoCrMo), and polypropylene (PP), were evaluated using five criteria: comfort and skin compatibility, elasticity, fatigue resistance, density, and energy dissipation. AHP was applied to determine the relative importance of the evaluation criteria using expert judgment, while VIKOR was used to rank the material alternatives and identify the compromise solution. The results showed that polyurethane achieved the best overall performance due to its balanced behavior in comfort, elasticity, and fatigue resistance, which are essential properties for long-term wearable neurorehabilitation devices. A sensitivity analysis confirmed that moderate variations in expert weighting did not modify the final ranking. Compared with conventional selection approaches based mainly on isolated material properties, the proposed framework offers a clear and reproducible method for integrating mechanical and user-related requirements into the material selection process for wearable orthoses.

## 1. Introduction

Neurorehabilitation aims to restore motor function and improve the quality of life for patients with neurological disorders, such as stroke, cerebral palsy, or spinal cord injury [1,2]. In recent years, wearable robotics has become an important tool in this field, supporting therapy by assisting movement, providing feedback, and enabling repetitive exercises that are difficult to achieve manually [3,4,5,6,7,8]. Among these devices, pressure-sensing orthoses have gained attention due to their ability to monitor force distribution and provide adaptive support to the user [9,10]. These orthoses are worn externally, do not require invasive procedures, and can be applied in both clinical and home settings. Their effectiveness depends not only on the mechanical design but also on the selection of materials that interact directly with the human body [11].

Material choice is critical for wearable pressure-sensing orthoses because the components in contact with the user must satisfy multiple functional and human-centered requirements [12]. Materials need to be sufficiently elastic to allow natural movement, durable to withstand repeated deformation, lightweight to minimize user fatigue, and comfortable against the skin to avoid irritation during extended use [13]. Additionally, energy dissipation properties influence the response of the system under cyclic loads, affecting both performance and comfort. Selecting materials that simultaneously satisfy all these requirements is a complex challenge, as improving one property may negatively affect another [14]. For example, materials with high elasticity may present lower fatigue resistance, while lightweight materials can be more susceptible to excessive deformation under load. These trade-offs make the design process complex and highlight the necessity of a structured evaluation approach [11,14].

**Figure 1:**
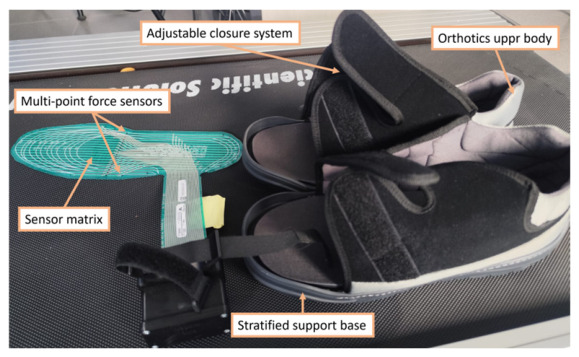
Plantar orthotic system with integrated pressure sensors.

The design of orthotic systems for neurorehabilitation requires a seamless integration of electronic components within the material structure of the device. As illustrated in Figure 1, the plantar orthotic system utilizes a sub-surface sensor matrix and multi-point force sensors to monitor pressure distribution in real time. This structural configuration emphasizes the importance of selecting materials that allow efficient force transmission while ensuring user comfort through active ventilation zones and adjustable closure systems [15,16].

The challenge of material selection is particularly relevant in the context of soft robotics [11]. Unlike rigid exoskeletons, soft robotic components must deform and conform to the anatomy of the user while maintaining mechanical integrity. Polymers, elastomers, and fabric-based composites are common candidates, each characterized by a combination of flexible strength, fatigue resistance, density, elasticity, and energy dissipation [17]. The interaction of these properties determines both device performance and user experience [15,16]. Despite advances in materials science, there is limited guidance on integrating these characteristics into a coherent decision-making framework for wearable pressure-sensing orthoses. Traditional approaches often focus on single material properties or empirical testing, which may not capture the multidimensional trade-offs required for optimal selection.

The problem is further complicated by the diverse environments and user needs in neurorehabilitation. Patients vary in body size, skin sensitivity, and mobility, while therapists require devices that are adaptable and reliable over repeated sessions. Materials that perform well under laboratory conditions may behave differently during prolonged daily use [18]. Similarly, orthoses used in home rehabilitation must maintain comfort and functionality without direct supervision, placing additional constraints on material properties. These considerations demonstrate that material selection is not purely a technical task but also a human-centered design problem that must balance functional performance and usability.

Given these challenges, multi-criteria decision-making (MCDM) methods offer a suitable approach for evaluating materials for wearable orthoses [19,20]. These methods allow multiple, often conflicting criteria to be considered simultaneously, providing a structured and transparent framework to support decision-making in complex engineering and resource management problems [21]. In the context of material selection for wearable pressure-sensing orthoses, multiple properties such as comfort, elasticity, fatigue resistance, density, and energy dissipation must be balanced, making MCDM particularly relevant [22].

This study adopts a hybrid approach that combines the Analytic Hierarchy Process (AHP) and the VIKOR (VlseKriterijumska Optimizacija I Kompromisno Resenje) method. AHP is used to determine the relative importance of the evaluation criteria through systematic pairwise comparisons, capturing expert judgment in a quantifiable way. VIKOR, on the other hand, focuses on ranking the material alternatives based on their performance across these weighted criteria, accounting for both the best-case and compromise solutions [23]. By combining these methods, the hybrid framework leverages the strengths of each: AHP provides a consistent and well-structured assessment of criteria importance, while VIKOR offers a compromise-oriented ranking that highlights alternatives closest to the ideal solution [19].

The hybrid use of AHP and VIKOR provides additional advantages compared to applying either method independently [19]. While AHP ensures that the weighting of criteria reflects expert knowledge and maintains logical consistency, VIKOR addresses the challenge of selecting among alternatives when criteria are conflicting and trade-offs are necessary [24]. Together, they create a comprehensive evaluation framework that captures both the relative importance of properties and the overall suitability of candidate materials [20]. This approach is particularly aligned with the objectives of this study, as it allows for an informed and systematic selection of materials that optimize both mechanical performance and user-centered considerations in wearable pressure-sensing orthoses.

In recent years, decision-support methods have been increasingly applied in biomedical engineering [20] and rehabilitation technology [19] to assist early-stage design processes where multiple technical and ergonomic factors must be evaluated simultaneously. In the case of wearable orthoses, material selection is often performed empirically or based on isolated mechanical properties, which can lead to inconsistent decisions between development teams or extended prototyping cycles. The use of MCDM methods allows engineering knowledge and clinical requirements to be organized into a structured evaluation process that can support both experienced designers and junior engineers during material selection. Within this context, the present study does not aim to fabricate or experimentally validate a complete orthotic prototype. Instead, it focuses on the development of a transparent decision-making framework intended to support the preliminary design stage of wearable pressure-sensing orthoses. The proposed approach provides a practical tool for comparing candidate materials before prototype manufacturing, helping reduce development time, material selection uncertainty, and unnecessary experimental iterations in wearable neurorehabilitation systems.

The objective of this research is to establish a structured framework for material selection in neurorehabilitation orthoses. The study focuses on materials that interact with the user’s body, considering properties such as comfort, elasticity, fatigue resistance, density, and energy dissipation. By determining the relative significance of these criteria and ranking potential materials, the research provides a quantitative basis for making informed material choices. This framework allows designers and engineers to address the trade-offs inherent in soft robotic components and supports decisions that optimize both functional performance and user comfort.

The results of this study are relevant to multiple stakeholders. Designers, engineers or project managers can use the framework to guide material selection during product development, ensuring that devices meet both technical and ergonomic requirements. Clinicians may benefit indirectly, as better material choices can enhance patient comfort, compliance, and therapy outcomes. Manufacturers and rehabilitation technology companies can apply the findings to streamline design processes, reduce prototyping costs, and improve product reliability. Additionally, the framework offers a replicable model for further research in wearable robotics, supporting systematic evaluation of materials in other soft robotic applications.

In summary, wearable pressure-sensing orthoses play an important role in neurorehabilitation, providing support and monitoring the patients with neurological impairments. Selecting materials for these devices is a complex task due to the need to balance multiple mechanical and human-centered criteria. This study applies a hybrid AHP-VIKOR approach to systematically evaluate and rank materials based on their performance across key criteria, providing a transparent and replicable framework for material selection. The outcomes contribute to the design of orthoses that are efficient, comfortable, and suitable for long-term use, supporting both clinical practice and technological development in neurorehabilitation.

## 2. Theoretical Framework

Wearable pressure-sensing orthoses are increasingly applied in neurorehabilitation to support motor recovery and monitor therapeutic progress [10,25]. These devices provide external support to the limbs, record force distribution, and assist in repetitive exercises without requiring invasive procedures [6,15]. While their mechanical design is critical for effective therapy, the performance and usability of orthoses also depend heavily on the selection of materials. Material properties influence comfort, durability, adaptability to body movements, and the overall effectiveness of neurorehabilitation interventions [26].

Selecting optimal materials for these devices is challenging because multiple criteria must be considered simultaneously. Factors such as elasticity, fatigue resistance, density, energy dissipation, and skin compatibility may interact in complex ways, and improvements in one characteristic can adversely affect others. The presence of such trade-offs needs a systematic evaluation approach capable of comparing materials across several performance dimensions [27].

Effective decision-making in this context requires defining two primary components: evaluation criteria and material alternatives. Criteria represent the key properties used to assess the suitability of materials in wearable pressure-sensing orthoses. Alternatives correspond to the candidate materials that can be used for constructing the device. A structured framework that incorporates both components ensures transparency, replicability, and rational prioritization in the material selection process.

### 2.1. Evaluation Criteria

The criteria used in this study were identified based on a review of scientific literature on soft robotics, wearable orthoses, and materials used in rehabilitation devices. Particular attention was given to properties that affect both device performance and user comfort. Five main criteria were selected for inclusion in the decision framework (Table 1).

These criteria together provide a balanced framework to evaluate the functional and ergonomic performance of candidate materials for wearable pressure-sensing orthoses. They reflect both human-centered considerations and mechanical requirements, ensuring that materials are assessed holistically.

### 2.2. Material Alternatives

The next step in the framework is to define the candidate materials to be evaluated. These materials will constitute the decision alternatives for the hybrid AHP-VIKOR assessment. While the specific alternatives (Table 2) will be selected based on availability, mechanical properties, and suitability for soft robotics applications, the framework allows for a systematic comparison once the alternatives are identified.

**Table 2:** Candidate materials considered in the decision framework.

Symbol	Material	Full Name	Description	Justification for Inclusion
A_1_	EVA	Ethylene Vinyl Acetate	A copolymer of ethylene and vinyl acetate characterized by low density, high flexibility, good shock absorption, and soft tactile properties. Commonly used in cushioning and wearable products.	Included due to its excellent comfort, flexibility, and widespread use in biomedical cushioning and orthotic padding, making it a relevant candidate for components in direct contact with the skin [32,33,34,35].
A_2_	PE	Polyethylene	A lightweight thermoplastic polymer with good chemical resistance, moderate stiffness, and low friction surface characteristics. Available in various grades such as LDPE and HDPE with different mechanical properties.	Selected because of its low density, biocompatibility, and frequent use in medical devices and flexible structural components, offering a balance between weight and mechanical stability [36,37,38].
A_3_	PU	Polyurethane	A versatile polymer that can be produced in rigid or highly elastic forms. It exhibits excellent abrasion resistance, high elasticity, and good fatigue performance under cyclic loading [39,40,41,42].	Chosen for its ability to withstand repeated mechanical stress and provide durability in joints or segments of the orthosis that require both flexibility and long-term resilience
A_4_	CoCrMo	Cobalt–Chromium–Molybdenum Alloy	A high-strength, corrosion-resistant metallic alloy commonly used in orthopedic implants. It provides excellent mechanical strength, wear resistance, and long-term structural stability [42,43].	Included as a candidate for structural load-bearing elements where superior mechanical integrity and biocompatibility are essential for the device’s framework.
A_5_	PP	Polypropylene	A semi-crystalline thermoplastic polymer with low density, good fatigue resistance, and moderate stiffness. It is widely used in lightweight structural components and medical-grade applications [32,44].	Selected due to its favorable strength-to-weight ratio and fatigue endurance, making it suitable for the rigid yet lightweight housing of wearable sensors.

By structuring the decision model around clearly defined criteria and alternatives, the theoretical framework establishes a solid foundation for applying multi-criteria decision-making methods. This framework ensures that both mechanical performance and user-centered considerations are integrated into the material selection process, supporting informed and consistent choices in the design of wearable pressure-sensing orthoses.

## 3. Materials and Methods

The selection of materials for wearable pressure-sensing orthoses involves balancing technical performance with patient comfort and long-term usability. In this study, a hybrid MCDM framework combining the AHP and VIKOR methods was adopted to support the early-stage material selection process. AHP was first applied to determine the relative importance of the evaluation criteria through structured pairwise comparisons based on expert judgment [19,20,24]. This step allows engineering experience and practical design considerations to be translated into quantitative weighting factors. Subsequently, the VIKOR method was used to rank the candidate materials by identifying a compromise solution that considers both overall performance and the limitations associated with conflicting criteria. Similar hybrid AHP-VIKOR approaches have been successfully applied in engineering and material selection problems where multiple mechanical and functional properties must be evaluated simultaneously. In the context of wearable orthoses, this methodology provides a transparent and reproducible decision-support tool that can assist designers, engineers, and project managers in comparing candidate materials before prototype fabrication or experimental testing. The combined approach also allows conflicting requirements, such as the trade-off between elasticity and fatigue resistance, to be evaluated through a structured mathematical process.

### 3.1. Determination of Criteria Weights Using AHP

The first stage of the decision process focuses on determining the relative importance of the selected material criteria. In the design of wearable pressure-sensing orthoses, different properties contribute unequally to the overall performance and usability of the device. For instance, parameters related to comfort and elasticity may be considered more critical than density, while fatigue resistance directly influences durability. Because these priorities cannot be measured directly, expert judgment must be translated into a consistent numerical form. The AHP was selected for this task due to its ability to structure subjective preferences and verify their logical consistency.

The set of evaluation criteria is defined as:

(1) $$C=\left(C_{1},C_{2},\;\ldots ,\;C_{n}\right)$$

Experts compare the criteria in pairs using Saaty’s fundamental scale, producing the pairwise comparison matrix:

(2) $$A=\left[a_{ij}\right]$$

where the element $a_{ij}$ represents the relative importance of criterion $C_{i}$ over criterion $C_{j}$. The matrix is reciprocal:

(3) $$a_{ij}=\frac{1}{a_{ij}}$$

This reciprocal structure reduces redundant judgments and ensures mathematical consistency in the comparison process.

Since the matrix contains relative rather than absolute values, normalization is required to transform the comparisons into comparable proportions. Each element is divided by the sum of its column:

(4) $${\tilde{a}}_{ij}=\frac{a_{ij}}{\sum_{k=1}^{n}{\tilde{a}}_{ij}}$$

The weight of each criterion is then obtained as the average of the normalized values across the corresponding row:

(5) $$\omega_{i}=\frac{1}{n}\sum_{j=1}^{n}{\tilde{a}}_{ij}$$

where $\omega_{i}$ denotes the final normalized weight of criterion $C_{i}$. These weights express the relative contribution of each material property to the overall evaluation.

Because expert judgments may contain inconsistencies, AHP incorporates a verification step. The consistency index is calculated based on the principal eigenvalue of the comparison matrix:

(6) $$CI=\frac{\lambda_{max}-n}{n-1}$$

The consistency ratio is then obtained as:

(7) $$CR=\frac{CI}{RI}$$

where RI is the random index corresponding to matrix size. A value of CR<0.1 indicates that the judgments are sufficiently coherent to be used in further analysis.

### 3.2. Ranking of Candidate Materials Using the VIKOR Method

After establishing the relative importance of the criteria, the next step is to evaluate and rank the candidate materials. The VIKOR method was selected for this phase because it identifies compromise solutions by simultaneously considering the overall performance of an alternative and its weakest criterion. This characteristic is particularly relevant for wearable orthoses, where poor performance in a single property may lead to discomfort or mechanical failure even if other properties are satisfactory.

The performance of the candidate materials across the selected criteria is represented by the decision matrix:

(8) $$X=\left[x_{ij}\right]$$

where $x_{ij}$ denotes the performance value of material i with respect to criterion j. These values are obtained from material datasheets and experimental measurements.

To evaluate how close each material is to the optimal solution, VIKOR defines the best and worst values observed for each criterion:

(9) $$f_{j}^{+}=\underset{i}{\max}x_{ij}$$

(10) $$f_{j}^{-}=\underset{j}{\min}x_{ij}$$

The best value $f_{j}^{+}$ represents the ideal performance, while $f_{j}^{-}$ represents the least desirable performance among the alternatives considered.

Two complementary measures are then calculated. The first, denoted as $S_{i}$, represents the aggregated distance of material i from the ideal solution across all criteria:

(11) $$S_{i}=\sum_{j=1}^{n}\omega_{j}\frac{f_{j}^{+}-x_{ij}}{f_{j}^{+}-f_{j}^{-}}$$

This measure reflects the overall performance of each material. The second measure, denoted as $R_{i}$, captures the maximum individual deviation from the ideal solution:

(12) $$R_{i}=\underset{j}{\max}\left[\frac{f_{j}^{+}-x_{ij}}{f_{j}^{+}-f_{j}^{-}}\right]$$

The $R_{i}$ value is essential in applications where a single critical weakness can compromise the functionality of the entire system.

The final compromise ranking index is calculated as:

(13) $$Q_{i}=v\frac{S_{i}-S^{+}}{S^{-}-S^{+}}+(1-v)\frac{R_{i}-R^{+}}{R^{-}-R^{+}}$$

where v is the strategy parameter expressing the relative importance of group utility versus individual regret. In this study, a balanced value of v = 0.5 is adopted to give equal importance to overall performance and worst-case behavior. Materials are ranked in ascending order of $Q_{i}$, with lower values indicating more suitable candidates.

**Figure 2:**
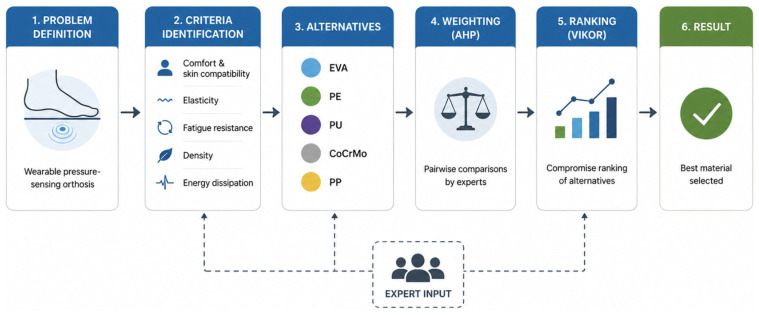
Workflow of current study.

Thus, the workflow presented in Figure 2 summarizes the sequence of steps followed for the evaluation and ranking of candidate materials used in wearable pressure-sensing orthoses.

Starting from the definition of the design problem and the selection of evaluation criteria, the process continues with the analysis of material alternatives through the AHP and VIKOR methods, leading to the identification of the most suitable material for neurorehabilitation applications. Figure 2 illustrates the complete methodological flow applied in this study.

## 4. Results

The implementation of the hybrid AHP-VIKOR model involved collecting expert judgments and processing the mechanical properties of the five materials: EVA, PE, PU, CoCrMo and PP. The selection of these candidates covers a wide spectrum of mechanical behaviors, ranging from highly compliant elastomers to high-strength metallic alloys, allowing the model to test the limits of its decision-making logic.

The first phase determined the importance of the five criteria: Comfort (C_1_), Elasticity (C_2_), Fatigue resistance (C_3_), Density (C_4_), and Hysteresis (C_5_). Based on the comparison matrix, the priority weights were calculated (Table 3). Comfort and skin compatibility emerged as the primary weight ($w_{1}$ = 0.378), followed by fatigue resistance ($w_{3}$ = 0.329). This weighting reflects a design philosophy where patient safety and device longevity take precedence over simple mass or energy efficiency. To ensure the results are valid, a consistency analysis was performed. The principal eigenvalue was found to be 5.38. With a matrix size of 5 and a random index of 1.12, the Consistency Index (CI) resulted in 0.095. The final Consistency Ratio (CR) was 0.0848. Because this value is below 0.1, the judgments are considered coherent and acceptable for the ranking phase.

The VIKOR method identified the best and worst values from the decision matrix. These points represent the ideal and least desirable performance for each criterion (Table 4). High R values, particularly evident in A_4_ and A_5_, indicate that these materials fail significantly in at least one priority area, such as comfort or elasticity, which is a major drawback for wearable applications.

**Table 4:** Ideal (best) and anti-ideal (worst) values for the orthotic material selection criteria.

Criterion	Best Value ($f_{j}^{+}$)	Worst Value ($f_{j}^{+}$)
C_1_ (Comfort and skin compatibility)	8.00	2.00
C_2_ (Elasticity)	8.33	1.25
C_3_ (Fatigue resistance)	9.00	5.33
C_4_ (Density)	8.33	1.25
C_5_ (Hysteresis)	8.50	4.33

Using these values, the group utility (S) and individual regret (R) were calculated (Table 5). The S value shows the overall distance to the ideal solution. The R value identifies the largest performance gap in a single criterion.

**Figure 3:**
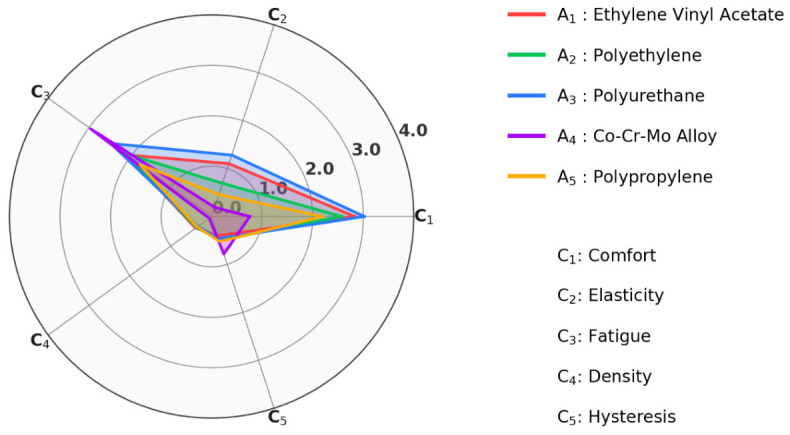
Normalized radar chart of material performance weighted by AHP importance.

The comparative performance of these materials is visualized in Figure 3. To provide an accurate representation of their suitability, the criteria axes in this radar chart have been normalized according to their relative weights established in the AHP phase. This normalization ensures that the area enclosed by each material’s plot directly reflects its weighted importance. Without this adjustment, criteria with different scales or lower significance would appear to contribute equally to the material’s total value. The resulting visualization clearly illustrates how materials like Polyurethane (A_3_) maximize performance across high-priority axes such as comfort and elasticity.

**Figure 4:**
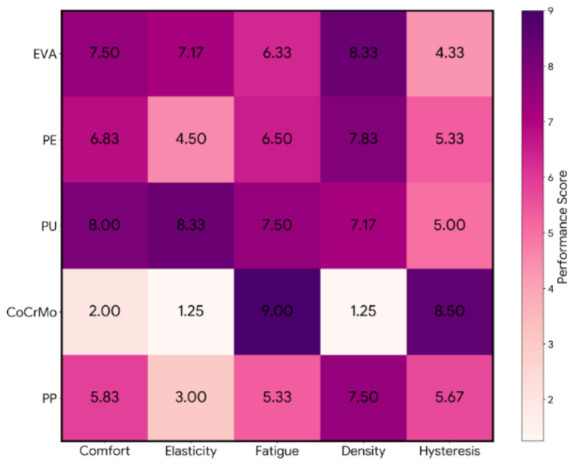
Heatmap of performance scores for material alternatives across multi-criteria indicators.

Before applying the weighting and ranking algorithms, it is essential to visualize the raw performance data of the selected materials across the identified criteria. Figure 4 presents the decision matrix in the form of a heat map, illustrating the performance scores for the five material alternatives: EVA, PE, PU, CoCrMo, and PP.

This visualization provides an immediate overview of the trade-offs inherent in material selection for neurorehabilitation. For instance, CoCrMo shows maximum values for Fatigue resistance, yet it scores significantly lower in Comfort and Density compared to polymer-based alternatives. Conversely, materials like PU and EVA exhibit high scores in ergonomic criteria, making them strong candidates for the soft interface of the orthosis. By mapping these values, the heat map serves as the foundational data set for the subsequent AHP-VIKOR analysis, ensuring that the final selection is based on a transparent and balanced evaluation of all mechanical and human-centered factors.

The final compromise ranking index (Q) was calculated using a strategy parameter of 0.5 (Table 6). This provides a balance between group utility and individual regret.

The final results show that Polyurethane (A_3_) is the most suitable material. It provides the best balance between durability and comfort for neurorehabilitation.

## 5. Sensitivity Analysis

A sensitivity analysis was carried out to examine the influence of expert weighting on the final material ranking obtained through the AHP framework. The analysis was performed in order to verify whether changes in expert importance could alter the ranking of the candidate materials.

The baseline case considered equal importance for all six experts:

(14) $$w_{i}=\frac{1}{6}=0.1667$$

The global score of each material was calculated using the weighted sum method:

(15) $$S_{j}=\sum_{k=1}^{5}W_{k}\cdot R_{jk}$$

where $S_{j}$ represents the global score of material j, $W_{k}$ is the weight of criterion k, and $R_{jk}$ is the aggregated rating of material j under criterion k.

The sensitivity analysis was performed using the One-at-a-Time approach. In each scenario, the weight of one expert was modified by ±20%, while the remaining expert weights were proportionally normalized. The modified weights were obtained using:

For the increase case:

(16) $$w_{i}^{new}=1.2\cdot w_{i}$$

For the decrease case:

(17) $$w_{i}^{new}=0.8\cdot w_{i}$$

The normalized weights were then computed as:

(18) $$w_{i}^{norm}=\frac{w_{i}^{new}}{\sum w_{i}^{new}}$$

The baseline global scores obtained for the candidate materials are presented in Table 7.

The same calculation procedure was repeated for all 12 perturbation scenarios corresponding to the increase and decrease in each expert weight.

The obtained results showed that the global scores and the final ranking remained unchanged in all investigated cases. Polyurethane (A3—PU) preserved the first position in every scenario, followed by EVA (A_1_), PE (A_2_), PP (A_5_), and CoCrMo (A_4_).

No ranking inversion was observed during the analysis. The results indicate that moderate variations in expert influence do not affect the final decision. Therefore, the proposed AHP framework shows stable behavior with respect to changes in expert weighting.

## 6. Discussion

The results of the hybrid AHP-VIKOR analysis indicate that polyurethane (PU, A_3_) achieved the best ranking with a compromise index Q value of 0.000. Ethylene vinyl acetate (EVA, A1) placed second at 0.4111, polyethylene (PE, A2) third at 0.457, polypropylene (PP, A5) fourth at 0.900, and cobalt–chromium–molybdenum alloy (CoCrMo, A4) last at 0.917. These positions follow directly from the AHP weights assigned to the criteria: comfort and skin compatibility at 0.378, fatigue resistance at 0.329, elasticity at 0.154, hysteresis at 0.092, and density at 0.047. In the decision matrix, PU recorded high scores of 8.000 on comfort, 8.330 on elasticity, and 7.500 on fatigue resistance. This balance produced low values for both group utility S (0.22) and individual regret R (0.135).

The ranking highlights the priorities for the soft interface layer in plantar pressure-sensing orthoses applied during neurorehabilitation. Patients recovering from stroke, cerebral palsy, or spinal cord injury wear these devices for extended sessions. Good skin compatibility helps avoid irritation and supports continued use of the therapy. Solid fatigue resistance allows the material to handle repeated loading from gait or assisted movements without fast loss of properties. PU met these needs across the criteria without large gaps.

Studies by Gerrard et al. [45] show that polyurethane, including PORON variants, reduces peak plantar pressure and peak force across all regions of the plantar foot compared to a shoe-only condition. Their systematic review found that polyurethane provided greater reductions in pressure-time integral than EVA in direct comparisons. Our model gave the highest weight to comfort and skin compatibility, and PU scored highest there while also performing well on fatigue. This outcome lines up with the pressure-reduction benefits reported for soft polyurethane foams in offloading applications.

Van Alsenoy et al. [46] examined custom foot orthoses made from EVA and expanded thermoplastic polyurethane (TPU, a close variant of PU) during running. They observed that TPU versions offered better resilience and maintained performance under repeated loading compared with EVA. In our decision matrix, PU scored higher than EVA on elasticity (8.330 versus 7.170) and fatigue resistance (7.500 versus 6.330), while EVA showed an advantage on density. The running studies noted that EVA can compress over longer use, which matches the lower fatigue score assigned to EVA here. TPU/PU materials resisted fatigue better, supporting the first-place position for PU when durability across many cycles matters for sensor-integrated devices.

Shi et al. [47] tested contoured insoles with different materials, including PORON Medical 4708 (a polyurethane foam) and Nora Lunalastik EVA, in elderly diabetic patients. They found that the soft polyurethane insole produced the lowest maximum plantar pressure and pressure-time integral values across toes, forefoot, midfoot, and rearfoot regions compared with EVA and more rigid materials. PORON Medical 4708 kept pressures below 200 kPa in most areas, a threshold linked to reduced risk of diabetic foot ulcers. Our comfort and fatigue scores for PU align with these findings, as the high performance on these criteria in our model favors materials that provide effective cushioning without rapid degradation.

Melia et al. [48] compared single-material softer polyurethane insoles with dual-material designs containing viscoelastic elements during loaded gait. The uniform soft PU insole reduced rearfoot and forefoot plantar pressure more effectively by increasing midfoot contact area. This supports the advantage shown by PU in our weighted evaluation, where it avoids weaknesses on high-priority criteria.

Rome [49] reviewed orthotic materials and noted that open-cell polyurethane foams show low compression set compared with some other foams when assessed alongside hardness and density. EVA provides lightweight cushioning but tends toward earlier compression under repeated cycles. Our AHP weights align with clinical priorities: comfort is the most important factor, as skin irritation can negatively affect patient compliance, followed by fatigue resistance to ensure reliable daily therapy without frequent replacement. Density received a low weight of 0.047 since minor mass differences matter less than overall function in lower-limb applications. The resulting ranking positions PU as the material that avoids major individual regret on the dominant criteria.

Some variation appears when the application changes. Salazar Loor et al. [22] applied TOPSIS, VIKOR, and COPRAS to orthotic splint design and selected polylactic acid (PLA) as best, largely for printability and balance in rigid or semi-rigid parts. Mian et al. [50] reached similar conclusions for knee orthoses, favoring PLA or PP where stiffness matters more. Our criteria set targets the soft sensor-interface layer, so elasticity and skin compatibility take priority and move polymers such as PU ahead. Another study on integrative MCDM for additive-manufactured orthoses also emphasized trade-offs and confirmed that no single material excels on every property.

Güner et al. [51] tested insoles with arch support, including polyurethane-based designs, in patients with multiple sclerosis. They observed changes in gait cycle parameters and improved sensory feedback in some areas, although overall gait impairments did not fully resolve. Their findings support the value of polyurethane for devices that combine pressure monitoring with comfort. Chen et al. reported that customized foot orthoses with layered designs redistribute plantar pressure and contact area, improving midfoot support. These outcomes align with the strengths shown by PU in our evaluation for sensor-integrated plantar applications.

On the technical side, hysteresis influences heat generation and sensor responsiveness during cyclic deformation. Our matrix assigned PU a moderate score of 5.000, better than EVA at 4.330. Running studies with EVA and TPU noted only small differences in economy, with more resilient materials helping limit increases in effort. The lower weight on hysteresis (0.092) prevented it from overriding the higher-priority comfort and fatigue criteria. The consistency ratio of 0.0848 from AHP shows the pairwise comparisons remained logically sound. VIKOR with a strategy coefficient of 0.5 balanced group utility against worst-case performance, which suits orthosis design where a single weak property can limit practical use.

Table 8 compares the performance scores from our study with key findings reported in the literature for the same or similar materials. Our scores come from the 1–9 scale in the decision matrix. Literature results include qualitative observations or quantitative measures such as pressure reductions or resilience notes. Citations appear inside the table cells where direct comparison data exist.

The table reveals clear agreement on soft polymers. Gerrard et al. [45] reported that polyurethane, polyethylene, and EVA all reduce plantar pressures, with polyurethane often showing stronger effects. Our weighted model ranks PU first because it balances fatigue and elasticity better for devices that must transmit force accurately to subsurface sensors while remaining comfortable. Studies by Van Alsenoy et al. [46] found EVA helpful for short-term cushioning and running economy, yet TPU variants handled repeated loading with less degradation. This pattern explains why PU received the lowest Q value while EVA placed second. Polypropylene receives attention in the literature mainly for rigid ankle-foot orthosis frames where its fatigue resistance under high loads proves useful [22,50]. Our lower ranking for PP fits the current focus on the soft plantar interface that needs to deform for sensor function and skin comfort. CoCrMo, widely studied for implants, delivers high fatigue and wear resistance but fails on comfort and elasticity, which matches why metals stay outside soft wearable layers.

In summary, the present results agree with published work showing that soft polymers such as PU and EVA offer effective pressure offloading and comfort in foot orthoses. Studies by Gerrard et al. [45], Shi et al. [47], and Van Alsenoy et al. [46] confirm the advantages of polyurethane variants for resilience and pressure reduction. The hybrid AHP-VIKOR model adds a quantitative layer by weighting criteria according to neurorehabilitation needs and producing a clear ranking for the soft interface. PU stands out as the best overall choice because it combines strong performance on the top-weighted properties without extreme weaknesses elsewhere. This selection supports reliable force transmission to sensors while promoting user comfort and device longevity during therapy.

The present study brings originality by applying the hybrid AHP-VIKOR method specifically to material selection for the soft skin-contact layer in plantar pressure-sensing orthoses for neurorehabilitation. Previous MCDM work in orthotics has often addressed rigid structural parts or general prosthetic decisions, using approaches such as TOPSIS, COPRAS, or standalone VIKOR for knee orthoses or 3D-printed components [22,50,53,55,56]. This research defines five criteria that address the combined requirements of accurate force transmission to subsurface sensors and prolonged comfort during wear. Energy dissipation receives separate treatment to capture hysteresis effects on dynamic response and heat buildup in gait cycles, an element less commonly isolated in standard comparisons of EVA, PU, and PE.

The set of candidate materials mixes everyday soft polymers with a metallic alloy to test how the model correctly places unsuitable options for deformable interfaces at the bottom. Expert-derived weights give comfort the leading position, which matches clinical experience where skin irritation quickly lowers therapy adherence. The framework combines datasheet values with measurements focused on cyclic loading and skin interaction. This produces a practical template that designers can update when new materials or patient requirements appear. Few studies connect MCDM directly to sensor-integrated wearable orthoses in the plantar area where the pressure mapping guides gait training for neurological patients. The approach therefore provides a transparent quantitative framework that goes beyond simple pairwise comparisons or single-property evaluations, enabling systematic material selection tailored to neurorehabilitation technology. The decision matrix relies on a restricted number of materials and performance values drawn from datasheets and controlled tests. Behavior under real conditions that include sweat, temperature shifts, and varying patient loads may differ from the laboratory data. Only five alternatives entered the evaluation; inclusion of additional foams, textiles, or composites could modify the order. Expert judgments for the AHP stage came from a small group working in rehabilitation engineering. Although the consistency ratio stayed acceptable, broader input from therapists and patients might adjust the weights, especially on comfort aspects. The model treats criteria as independent even though properties such as elasticity and hysteresis interact during actual dynamic use. VIKOR applied a balanced strategy coefficient of 0.5; other values could change the emphasis according to particular device goals. The analysis covers the soft interface layer solely. Complete orthosis systems also include frames, closures, and electronics that call for separate review. Cost and ease of manufacturing did not enter the criteria, yet these factors influence decisions when production scales up. Long-term trials with patients remain necessary to verify gains in compliance, sensor data quality, and clinical results over months of daily application.

The available research provides several sets of experimental data and numerical simulations focused on the ability of polymer and composite materials to redistribute plantar pressures (Table 9). These studies evaluate how different materials improve biomechanical comfort when used in orthotic devices and top covers [47].

**Table 9:** Overview of research data on biomechanical comfort and plantar pressure redistribution in polymer-based orthotic devices.

References	Materials Evaluated	Results/Key Findings
Effects of contoured insoles with different materials on plantar pressure offloading in diabetic elderly during gait [47]	EVA (Nora Lunalastik), Nora Lunalight A fresh, Pe-Lite (PE), PORON (PU)	PORON Medical 4708 (PU) produced the lowest mean peak pressure (MPP) and pressure-time integral (PTI) across all foot regions. The data showed that soft materials performed better than rigid options for pressure relief.
The Effect of EVA and TPU Custom Foot Orthoses on Running Economy, Running Mechanics, and Comfort [46]	EVA, expanded TPU (Thermoplastic Polyurethane)	EVA custom orthoses reduced braking force by 3.4% at low speeds. Both EVA and TPU materials improved perceived comfort levels regarding arch height and lateral control during physical activity.
The effect of PORON and Plastazote insoles on forefoot plantar pressures [55]	PORON (PU), PORON + Plastazote (PE)	Both PORON and PORON/Plastazote insoles lowered peak pressures under the forefoot. Testing indicated that PORON peak pressures increased after 50,000 steps because the material experienced mechanical fatigue.
Preliminary investigation on the reduction in plantar loading pressure with different insole materials [56]	Poron (PU), Plastazote (PE), PORON + Plastazote (firm & soft)	The combination of Poron and firm Plastazote (PPF) led to a 27% reduction in mean pressure compared to barefoot conditions. All tested PU materials decreased pressure levels across the entire plantar surface.
Foot plantar pressure offloading: how to select the right material for a custom-made insole [57]	Soft materials vs. Stiff materials	Pressure offloading is achieved by placing soft materials under areas with high pressure and using stiffer materials in other sections. FEM simulations predicted these pressure changes accurately before production.

The experimental data gathered from these studies show that PU-based materials are effective at reducing peak pressure points. At the same time, hybrid combinations using PE or EVA remain useful for maintaining long-term structural stability and durability.

The assessment of the proposed methodology through a SWOT analysis (Table 10) provides a meaningful perspective on how the AHP–VIKOR hybrid framework functions within the field of material science for neurorehabilitation. While this decision-making tool brings significant contributions by organizing complex data into a logical structure, it is equally important to acknowledge that any theoretical model comes with both practical benefits and certain boundaries. Evaluating these internal and external factors helps in understanding the overall value of the work and identifies the specific areas where the approach succeeds or requires further refinement.

**Table 10:** Material ranking under expert weight perturbation scenarios.

**Strengths**	**Weaknesses**
The method changes a subjective decision process into a structured and logical path.	The study depends on theoretical data and the opinions of a specific group of experts.
It balances conflicting factors like cost, durability, and patient comfort.	There is a lack of physical testing on a final prototype to confirm the material behavior.
The mathematical foundation reduces human error in the ranking process.	The math involved can be difficult to handle without specialized tools.
**Opportunities**	**Threats**
The system can grow by including direct feedback from medical staff to improve the weighting.	Fast development of new materials might offer cheaper ways to get similar results.
There are numerous studies exploring new materials, and this model can be used to test them.	Other decision models might be simpler to use for small-scale projects.
It can be adapted for other types of wearable medical devices.	Changes in medical regulations could require new and different criteria.

A primary strength of this approach is the way it successfully handles several factors that often conflict. By using this mathematical foundation, the choice of PU is supported by data. However, a present weakness is the heavy reliance on the opinions of experts. This creates a gap because the work does not yet include experimental validation to confirm how the material behaves during actual use.

There are good opportunities to improve this system in the future by adding a wider variety of materials to the evaluation list, especially since many recent works are constantly proposing new options. At the same time, the rapid appearance of these materials is a threat, as they might offer different advantages. Understanding these points shows that the current framework is a solid tool for design, but its practical value will be fully proven once physical testing is finished.

Future steps should involve fabrication of prototypes that use PU as the main interface material together with the sensor matrix. Direct testing would measure pressure distribution accuracy, comfort ratings from users, and durability across daily gait cycles. Parallel trials with EVA-based versions could quantify differences in fatigue levels and therapy adherence for targeted patient groups such as those with stroke or cerebral palsy. The material database can grow to cover advanced elastomers, smart textiles, or new printable options to examine how well the model holds up. Weights may receive updates through wider surveys that include stakeholders across impairment types and age ranges. Methods for managing uncertainty, including fuzzy versions of AHP or interval-based VIKOR, would handle variation in supplier data more effectively. Longitudinal observations should track material degradation, sensor signal stability, and skin health indicators over extended periods. Integration with machine learning could let the framework suggest materials based on data gathered directly from the orthosis during use. Hybrid constructions that combine PU with improved ventilation or antimicrobial layers also warrant investigation. Extension of the same framework to upper-limb or spinal orthoses would increase its value across neurorehabilitation technologies.

## 7. Conclusions

This study developed and applied a hybrid multi-criteria decision-making framework combining AHP and VIKOR to select the optimal material for the soft skin-contact layer in plantar pressure-sensing orthoses intended for neurorehabilitation. Five candidate materials were systematically evaluated according to five relevant criteria that address both functional performance and user comfort.

The analysis identified PU as the most suitable material, offering the best compromise solution across the evaluated criteria. It demonstrated a balanced performance that satisfies the primary requirements of comfort, durability under repeated loading, and adaptability to body movements. The ranking placed the remaining materials in descending order of suitability, clearly highlighting the limitations of stiffer options for applications requiring direct skin contact and sensor integration.

These findings align with previous research on orthotic materials, which consistently shows that PU provides effective pressure redistribution and superior resilience compared with EVA under cyclic conditions. The results further confirm that materials such as PP and metallic alloys, although strong in structural applications, are less appropriate for the soft interface layer due to inadequate comfort and elasticity.

The hybrid AHP-VIKOR approach proved effective in managing trade-offs between conflicting criteria and delivered a transparent, reproducible decision process. By prioritizing expert judgment in weighting and compromise ranking, the framework avoids over-reliance on single properties and supports more informed material choices.

The outcomes of this work are relevant to multiple stakeholders. Designers and engineers can apply the proposed methodology in the early stages of device development to reduce prototyping iterations and accelerate the design process. Clinicians and therapists may benefit from devices that offer better patient compliance and more consistent performance during rehabilitation sessions. Manufacturers of neurorehabilitation technology can use the framework to optimize material selection, improve product reliability, and facilitate scaling toward home-based applications. Furthermore, future research should incorporate cost-effectiveness analyses and resource consumption assessments to evaluate the economic viability and sustainability of the proposed materials for large-scale production and widespread clinical adoption.

Overall, this study contributes a practical decision-support tool tailored to sensor-integrated soft orthoses. It addresses the specific challenges of wearable robotics in neurorehabilitation by focusing on the critical interface between the device, the sensors, and the user’s skin. The methodology can be readily adapted to other soft robotic components where multiple mechanical and human-centered requirements must be balanced simultaneously.

In conclusion, the hybrid AHP-VIKOR framework successfully identified PU as the preferred material for the soft interface of plantar pressure-sensing orthoses. This selection supports the development of more comfortable, durable, and effective assistive devices that can enhance motor recovery and improve quality of life for patients with neurological disorders.

## Figures and Tables

**Table 1:** Evaluation criteria for material selection in wearable pressure-sensing orthoses.

Symbol	Criteria	Description	Justification for Inclusion
C_1_	Comfort and skin compatibility	Ability of the material to be in contact with the skin for long periods without causing irritation, allergic reactions, excessive heat, or moisture accumulation.	Essential for devices that are worn daily, affecting user compliance and therapy outcomes [27,28].
C_2_	Elasticity	Capacity of the material to deform under load and return to its original shape after removal of the force.	Influences freedom of movement, adaptability to the user’s anatomy, and force transmission efficiency in soft robotic components [28,29,30].
C_3_	Fatigue resistance	Ability of the material to withstand repeated cycles of loading and unloading without degradation, cracks, or loss of mechanical properties.	Critical for components that experience continuous deformation during rehabilitation exercises [10,25].
C_4_	Density	Mass per unit volume of the material.	Affects overall weight of the orthosis, user effort, and energy consumption during long-term use [26,30,31].
C_5_	Energy dissipation (hysteresis)	Loss of energy during a complete deformation-recovery cycle.	Materials with high hysteresis dissipate more energy as heat, which may reduce mechanical efficiency and dynamic response [26,29,30].

**Table 3:** Decision matrix with criteria weights obtained through the AHP method.

Material	C_1_ (Comfort and Skin Compatibility) ($w_{1}$ = 0.378)	C_2_ (Elasticity) ($w_{2}$ = 0.154)	C_3_ (Fatigue Resistance) ($w_{3}$ = 0.329)	C_4_ (Density) ($w_{4}$ = 0.047)	C_5_ (Hysteresis) ($w_{5}$ = 0.092)
A_1_—EVA	7.500	7.170	6.330	8.330	4.330
A_2_—PE	6.830	4.500	6.500	7.830	5.330
A_3_—PU	8.000	8.330	7.500	7.170	5.000
A_4_—CoCrMo	2.000	1.250	9.000	1.250	8.500
A_5_—PP	5.830	3.000	5.330	7.500	5.670

**Table 5:** Calculated values for group utility and individual regret for each candidate material.

Criterion	Group Utility (S)	Individual Regret (R)
A_1_—EVA	0.389	0.239
A_2_—PE	0.455	0.224
A_3_—PU	0.22	0.135
A_4_—CoCrMo	0.578	0.378
A_5_—PP	0.65	0.329

**Table 6:** Final VIKOR ranking and compromise solution (Q values) for a consensus strategy.

Material	Q Value	Rank
A_3_—PU	0.000	1
A_1_—EVA	0.4111	2
A_2_—PE	0.457	3
A_5_—PP	0.900	4
A_4_—CoCrMo	0.917	5

**Table 7:** Global scores and ranking of alternatives.

Material	Global Score	Rank
A_3_—PU	7.571	1
A_1_—EVA	6.812	2
A_2_—PE	6.272	3
A_5_—PP	5.293	4
A_4_—CoCrMo	4.750	5

**Table 8:** Comparative performance of materials: our AHP-VIKOR results versus literature findings.

Material	Comfort Score	Literature on Comfort/Plantar Pressure Reduction	Fatigue Score	Literature on Durability/Fatigue	Elasticity Score	Literature on Elasticity/Resilience
A_3_—PU	8	High reductions in peak pressure and pressure-time integral; PORON PU best offloading with lowest maximum pressures [47]; greater reductions than EVA [45]; effective cushioning in diabetic patients [48]	7.5	Minimal compression set; better resilience under cyclic loading than EVA [46]; low compression set in open-cell PU foams [49]	8.33	High resilience in TPU; maintains performance across repeated tasks [46]; higher rebound than EVA [46]
A_1_—EVA	7.5	Good pressure reduction, especially under heel and forefoot; improves short-term perceived comfort [46]; effective but higher pressures than PU; reduces total plantar pressures in specific activities [52, 53, 54]	6.33	Shows compression set over time; resilience around 37% in ball rebound tests [46]; earlier compression under repeated cycles [49]	7.17	Flexible but lower long-term resilience compared with TPU; moderate flexibility in cushioning layers [45]
A_2_—PE	6.83	Effective pressure reduction similar to PU and EVA [45]; good for lightweight cushioning layers	6.5	Moderate durability; used in layers but less resilient under heavy cycles [45]; earlier compression set than PU [49]	4.5	Moderate flexibility; suitable for cushioning layers [45]
A_5_—PP	5.83	Moderate in soft interfaces; stronger for rigid structural shells [22,50]	5.33	Good fatigue resistance in load-bearing frames but stiffer overall [22] materials review [13])	3	Lower elasticity; preferred for frames rather than deformable skin-contact layers [22,50]
A_4_—CoCrMo	2	Not used in soft orthoses; poor skin compatibility despite high strength in implants	9	Excellent wear resistance in metallic implants	1.25	Very low; rigid nature unsuitable for deformation and comfort needs

## Data Availability

The data that support the findings of this study are available from the corresponding author upon reasonable request.
